# The Curious Case of Impersonators and Singers: Telling Voices Apart and Telling Voices Together under Naturally Challenging Listening Conditions

**DOI:** 10.3390/brainsci13020358

**Published:** 2023-02-19

**Authors:** Sarah V. Stevenage, Lucy Singh, Pru Dixey

**Affiliations:** School of Psychology, University of Southampton, Southampton SO17 1BJ, UK

**Keywords:** voice identity, telling voices together and apart, impersonator voices, singing voices, natural listening challenge, identity regions

## Abstract

Vocal identity processing depends on the ability to *tell apart* two instances of different speakers whilst also being able to *tell together* two instances of the same speaker. Whilst previous research has examined these voice processing capabilities under relatively common listening conditions, it has not yet tested the limits of these capabilities. Here, two studies are presented that employ challenging listening tasks to determine just how good we are at these voice processing tasks. In Experiment 1, 54 university students were asked to distinguish between very similar sounding, yet different speakers (celebrity targets and their impersonators). Participants completed a ‘Same/Different’ task and a ‘Which is the Celebrity?’ task to pairs of speakers, and a ‘Real or Not?’ task to individual speakers. In Experiment 2, a separate group of 40 university students was asked to pair very different sounding instances of the same speakers (speaking and singing). Participants were presented with an array of voice clips and completed a ‘Pairs Task’ as a variant of the more traditional voice sorting task. The results of Experiment 1 suggested that significantly more mistakes were made when distinguishing celebrity targets from their impersonators than when distinguishing the same targets from control voices. Nevertheless, listeners were significantly better than chance in all three tasks despite the challenge. Similarly, the results of Experiment 2 suggested that it was significantly more difficult to pair singing and speaking clips than to pair two speaking clips, particularly when the speakers were unfamiliar. Again, however, the performance was significantly above zero, and was again better than chance in a cautious comparison. Taken together, the results suggest that vocal identity processing is a highly adaptable task, assisted by familiarity with the speaker. However, the fact that performance remained above chance in all tasks suggests that we had not reached the limit of our listeners’ capability, despite the considerable listening challenges introduced. We conclude that voice processing is far better than previous research might have presumed.

## 1. Introduction

The last thirty years has seen a growing awareness of the voice as a valuable cue to identity [1,2]. This has fuelled a considerable research effort aimed towards a greater understanding of voice processing. In particular, researchers have considered the parallels that may exist between voice processing and face processing, with each combining to give a multimodal perspective on person perception [3]. Along these lines, Belin, Fecteau, and Bédard [4] came to view the voice as an ‘auditory face’ and described in cognitive terms, and later in neuropsychological terms [5], how voice and face processing contribute to the analysis of speech and also affect identity. This framework accommodated the importance of both unimodal and multimodal processing for both voice and face identification [6]. It also served as a guide for a body of work that applied face recognition methodologies to the voice recognition domain as a way to determine just how good the voice recognition was.

### 1.1. Voice Recognition Is a Challenging Task

The work that followed indicated that voice recognition was challenging compared to face recognition. Early studies showed that there were more familiar-only experiences [7,8] and more tip-of-the-tongue states [7] when presented with a voice than when presented with a face. Similarly, voices were less likely than faces to elicit the retrieval of episodic information such as details of the last encounter [9,10,11]. Voices were also less likely than faces to elicit the retrieval of semantic information such as a person’s occupation [8,10,12,13,14]. Finally, in what has become known as the facial overshadowing effect, subsequent voice recognition was worse when voices and faces were initially presented together at study, than when voices were initially presented alone at study [15,16,17,18]. Together, these findings suggest that the pathway supporting voice identity processing is weaker than that supporting face identity processing. This said, similarities can be drawn between voice and face identity processing.

### 1.2. Recognition, Telling Together and Telling Apart

Recognising a face or a voice relies on the ability to map an exemplar onto a stored mental representation. That stored mental representation may be viewed as a point in some multidimensional space, with recognition occurring when an individual exemplar is mapped closer to its stored mental representation than to any other stored mental representation [19,20]. Equally, however, the stored mental representation may be viewed as a region within multidimensional space, and this view captures the fact that the natural variation within a person is as important as the variation between people. Indeed, when one considers the fact that an individual’s face or voice can change from moment to moment, then it becomes clear that the recognition task is as much about spotting the similarities between instances of the same person as it is about spotting the differences between instances of two different people [21].

Inventive new methodologies have been developed to explore face and voice recognition from the perspective of spotting similarities and differences within and across targets. Notable amongst these is the sorting task, first used with faces [22], and then adapted for voices [23]. This work revealed that participants were better able to tell people apart than they were to tell people together. That is, when mistakes were made, they tended to result in two instances of the same person being mistakenly judged as two different people. This error was, however, far less likely if the targets were familiar. Specifically, Lavan, Burston, and Garrido [23] used voices and asked listeners to sort 30 sound clips taken from pairs of characters from the TV series ‘Orange is the New Black’. By listening to the clips and dragging them together to form identity clusters on a PowerPoint slide, listeners were able to reveal the number of identities that they believed were contained in the set. Unfamiliar listeners who had never watched the TV show believed there to be a mode of 6.67 identities (range = 2–17 identities), whereas familiar participants were in line with the truth, believing there to be a mode of two identities (range = 2–12 identities) (see [24] for similar results). Familiarity gave the listeners an advantage, principally because they could better spot similarities between the clips from the same speakers.

In a subsequent study, Lavan, Burston, Ladwa, Merriman, Knight, and McGettigan [25] challenged their listeners to see whether they were still able to sort voices into identities when the voices differed to a greater degree through increased expressiveness. Using 30 highly expressive and 30 lowly expressive voice clips from the two main characters of the TV series ‘Breaking Bad’, participants again completed a sorting task as above. Unfamiliar listeners who had never watched the show sorted the 30 lowly expressive clips into a mode of six identities (range = 3–16 identities), whereas familiar listeners were again closer to the truth with a mode of three identities (range = 2–9 identities). These results thus replicated the familiarity advantage noted previously. In contrast, high vocal expressivity presented a particular challenge to the unfamiliar listeners. They perceived a mode of nine identities (range = 4–15 identities) whilst the familiar listeners perceived a mode of two identities (range = 2–9 identities). This said, both listener groups showed more confusion errors when sorting the highly expressive voices by muddling speakers within the same identity cluster. This showed the difficulty imposed by high expressivity in the voice, arguably because the two different speakers could have sounded more similar to one another in the highly expressive clips than in conversational speech. Nevertheless, the listeners were still able to sort the highly expressive voices, particularly when familiar with them.

### 1.3. The Present Studies

What is clear from the preceding discussion is that voice recognition is a relatively difficult task compared to face recognition, and that spotting similarities between different instances of the same person appears to be more difficult than spotting differences between similar sounding instances of different people. However, whilst challenges have been levelled at the listener in preceding studies, we have not yet defined the limits of our voice recognition capability. Consequently, two questions remain to be addressed: First, how similar must clips of two people’s voices be for us to fail to tell them apart? Second, how different must clips of a single voice be for us to fail to tell them together? Two experiments were designed to address these questions with the goal of defining the limits of normal voice processing.

## 2. Experiment 1: Telling Apart Voices of Celebrities and Their Impersonators

Vocal impersonation occurs when one speaker tries to sound like another. This is most commonly for the purpose of humour or entertainment, but impersonation can present a problem if the listener is fooled by the impersonator. This represents a failure to tell apart the voices of two similar sounding yet different speakers. Vocal impersonators thus provide an excellent means of studying this challenge because they present a natural yet extreme level of similarity for the listener to resolve. (Note that the literature refers to ‘vocal imitation’ and ‘vocal conversion’ as well as ‘vocal impersonation’. For consistency, the term ‘impersonation’ is used throughout this manuscript.)

The voices of impersonators have been examined in only a few studies and most of these have concentrated on the vocal parameters that the impersonator changed in order to sound like their target. For instance, when impersonating their target, one professional Swedish impersonator changed prosodic features including pitch, timing of vowel sounds, and speaking rate. They also used words and phrases characteristic of the target in order to capture idiosyncratic vowel sounds (as reflected in formant frequencies) and to influence the readiness of listeners to accept the impersonation [26]. Similarly, one Japanese impersonator changed their pitch and pitch frequency contour to make their voice higher pitched and more melodic, but they were also able to change glottal characteristics to make their voice hoarser [27]. Interestingly, Zetterholm [28] demonstrated that different impersonators tended to pick out the same prominent features of a given target. She also showed that impersonators at times exaggerated or caricatured these features rather than producing a faithful replica (see [26,27,28,29]).

Less common within the literature is research on the extent to which listeners may be fooled by these impersonators. In this regard, López, Riera, Assaneo, Eguía, Sigman, and Trevisan [30] presented some useful insights. They used the impersonations of three targets by five professional impersonators, distinguishing between impersonations uttered from memory (which they called ‘caricatures’) and those uttered following presentation of the target as a guide (which they called ‘replicas’). The replicas generated a greater sense of likeness to the target (than did the caricatures) in a similarity rating task. Interestingly, however, and of more pertinence to the current study, the caricatures generated a greater sense of belonging to the target (than did the replicas) in an identity rating task, with two of the impersonators being mistaken for two of the targets. This suggests a failure to distinguish these targets from their impersonators.

Experiment 1 provides an extension of the work by López et al. [30] with a more extensive set of impersonators and targets, and an expanded set of tasks. Of primary interest was whether the listeners would be fooled by the impersonators or whether they would succeed in telling apart the targets and impersonators despite the natural challenge being presented.

## 3. Materials and Methods—Experiment 1

### 3.1. Design

The participants completed three separate tasks within Experiment 1, each of which tested their capacity to discriminate between celebrity targets and their impersonators. Task 1 was a ‘Same/Different’ task to pairs of stimuli, with pair type (target–target, target–impersonator, target–other) representing the independent variable. This thus represented a perceptual discrimination task without explicitly needing to know the identity of the speaker. Task 2 was a ‘Real or Not?’ task to individual stimuli, with identity (target, impersonator, other) representing the independent variable. This represented a recognition task by requiring a comparison of the sample to some stored mental representation. Finally, Task 3 was a ‘Which is the Celebrity?’ task to pairs of stimuli, with pair type (target–impersonator, target–other) representing the independent variable. This required a comparison of each sample to a stored mental representation in order to choose which represented the named target. The accuracy of performance for the targets, impersonators, and other voices represented the dependent variable in each task.

### 3.2. Participants

A total of 54 participants (14 males, 33 females, seven undeclared) took part on a volunteer basis or were recruited via a departmental participant scheme and took part in return for course credit. The sample size was determined by availability, however, a post-hoc power calculation using G*Power 3.1.9.7 (with alpha set to 0.05) showed that our sample size was associated with a power of no less than 0.9998 across the three tasks.

The participant ages ranged from 18 to 29 years (mean age: 21 years, SD = 2.09) and all participants had normal, or corrected-to-normal hearing as determined through self-report. They also reported a good knowledge of current affairs from which the targets were drawn.

### 3.3. Materials

#### 3.3.1. Celebrity Targets

A total of 84 speech clips were obtained from 12 celebrity speakers (seven clips per speaker). All celebrities (eight males, four females) were drawn from stage, screen, and politics. To maintain anonymity, celebrities are denoted by their initials throughout this manuscript. The clips were extracted from longer segments from chat shows or radio interviews, and care was taken to ensure that the seven clips per speaker were drawn from at least two separate interviews and thus could not be matched based on incidental features such as background noise or production quality. Additionally, clips from the same interview did not run consecutively from one another, ensuring that they could not be matched based on speech content.

#### 3.3.2. Impersonators

A total of 36 speech clips were obtained from six professional impersonators who together provided three compelling impersonations for each of the 12 celebrity targets. As above, these were extracted from longer segments available online. By their nature, the impersonators spoke about similar semantic themes as the targets they impersonated.

#### 3.3.3. ‘Matched Other’ Voices

A total of 36 clips were obtained from 12 other celebrity speakers (three clips per speaker). These speakers were matched to the celebrity targets on sex, broad age category, and broad accent. They were not, however, trying to sound like the target celebrities. Their inclusion made it possible to determine the baseline levels of voice discrimination when presented with a pair of different speakers. However, their broad matching ensured that the baseline discrimination task was a non-trivial task.

#### 3.3.4. Editing of Clips

All voice clips were edited using Audacity 3.1.0 to produce complete phrases lasting 3–8 s. All clips were normalised in terms of volume and the preparation of multiple clips ensured that no clip was repeated in the entire set of tasks.

#### 3.3.5. Trial Construction

The ‘Same/Different’ task consisted of 36 trials in which pairs of voice clips were presented. For 12 trials, two clips of each target speaker were presented, and thus the correct answer was ‘same’. For a further 12 trials, each target speaker was presented alongside their impersonator. The correct answer was thus ‘different’, but this represented a difficult discrimination task. Finally, for a further 12 trials, each target speaker was presented alongside their ‘matched other’ celebrity. The correct answer was thus ‘different’, and this tapped the baseline levels of discrimination.

The ‘Real or Not?’ task consisted of 36 trials in which single voice clips were presented alongside a target name. These 36 trials consisted of the presentation of the 12 targets for whom the correct answer was ‘real’. Alongside these, the participants were also presented with the 12 impersonators for whom the correct answer was ‘not real’, but the task was difficult. Finally, the task included the 12 ‘matched other’ celebrities for whom the correct answer was ‘not real’, and this tapped the baseline levels of recognition.

Finally, the ‘Which is the Celebrity?’ task consisted of 24 trials in which pairs of voice clips were presented along with a target name. For 12 trials, a clip of the real target was presented alongside their impersonator, representing a difficult discrimination task. For the remaining 12 trials, a clip of the real target was presented alongside the ‘matched other’ celebrity representing the baseline discrimination task.

#### 3.3.6. Presentation of Stimuli

The stimuli were presented and data recorded using bespoke survey software (isurvey.soton.ac.uk) run on a Dell Latitude E7250 laptop with an Intel Core i5 vPro processor and 8 GB of RAM. Voice clips were played via the laptop speakers set to an audible but adjustable level.

### 3.4. Procedure

The participants were tested individually and in-person. Following the provision of informed consent and the completion of a sound check within which they could adjust the volume to suit, the participants completed the three tasks in one of two set orders. Given that the ‘Which is the Celebrity?’ task explicitly revealed the fact that impersonators were being used, its early completion may have changed the way that the participants listened to the voices in the remaining tasks. Consequently, all participants completed the ‘Which is the Celebrity?’ task last. However, the participants were randomly assigned to either receive the ‘Same/Different’ task first or the ‘Real or Not?’ task first. This aside, the procedure within each task was identical regardless of their order, and the onscreen instructions prepared the participant for each task.

In the ‘Same/Different’ task, the participants were presented with pairs of voice clips featuring either the target (‘same’ trial), the target and impersonator (difficult ‘different’ trial), or the target and the ‘matched other’ celebrity (baseline ‘different’ trial). Participants were instructed to click on icons to play the clip, and they could replay these as many times as required. After hearing the clips, they were then asked whether the second clip was spoken by the same speaker or a different speaker compared to the first clip. Participants responded ‘same’ or ‘different’ by clicking on one of two onscreen buttons. The 36 trials were presented in a random order, and participants were encouraged to respond as accurately as possible. No time pressure was imposed, and no feedback was provided.

In the ‘Real or Not?’ task, participants were presented with a single voice clip alongside the question ‘Is this …?’ The question provided the name of a target celebrity, and the voice clip was either the celebrity, the impersonator, or the ‘matched other’ celebrity. Participants clicked on an icon to hear the clip and could replay it as many times as required before responding ‘real’ or ‘not real’ by clicking on one of two onscreen buttons. As above, the 36 trials were presented in a random order, accuracy was encouraged, no time pressure was imposed, and no feedback was provided.

In the ‘Which is the Celebrity?’ task, participants were presented with two voice clips labelled Voice A and Voice B alongside the question ‘Which of these voices is …?’ The question provided the name of a target celebrity and the voice pairs either represented the target and their impersonator, or the target and their ‘matched other’ celebrity. Participants clicked on the icons to play each voice clip and could replay these as required. Then, the participants clicked on one of two onscreen buttons to choose either voice A or voice B as the named celebrity. The 24 trials were presented in a random order. As above, accuracy was encouraged, no time pressure was imposed, and no feedback was provided.

Following the completion of all three tasks, participants completed a familiarity check in response to the names of all 12 targets plus the 12 ‘matched other’ celebrities. Participants were asked to rate each person from 1 to 7, with a rating of 1 indicating that they did not know the person at all, and a rating of 2–7 indicated the level of familiarity with a known person. This rating procedure thus allowed the possibility to remove trials in the ‘Real or Not?’ task and the ‘Which is the Celebrity?’ task, where celebrities were unknown (rating of 1) and could not have been recognised.

The entire procedure lasted about 40 min after which the participants were thanked and debriefed.

### 3.5. Data Management and Statistical Analysis

Data were collected in accordance with ethical principles as laid out in the Declaration of Helsinki. In particular, informed consent was obtained and all data were pseudo-anonymised, enabling participants to exercise the right to withdraw. Anonymous summary datafiles are available and can be downloaded from the Supplementary Materials.

Statistical analyses were conducted to test whether the challenge of distinguishing celebrity targets from their impersonators represented an impossible task. Preliminary investigation using Kolmogorov–Smirnov tests suggested that the accuracy scores in all three tasks were not normally distributed (*D* > 0.126, *p* < 0.036). This, plus the relatively small sample size in Experiment 1, suggested the use of non-parametric statistical tests. Consequently, Friedman’s analyses of variance were used to examine the performance across experimental conditions, with Wilcoxon tests used for follow-up comparisons. An alpha of 0.05 was assumed throughout, with Bonferroni-correction in the case of multiple comparisons.

## 4. Results—Experiment 1

The familiarity check confirmed that all participants were familiar with all targets. Thus, no individual trials were dropped for individual participants. Following this, the mean accuracy was calculated for each trial type in each of the three experimental tasks. The data from two participants were removed from the ‘Same/Different’ task and the data from one participant were removed from the ‘Real or Not?’ task through being identified as outliers (below 1st quartile minus 1.5 × interquartile range). This, combined with dropout prior to the last task, resulted in the analysis of data from 51, 52, and 48 participants in the ‘Same/Different’ task, the ‘Real or Not?’ task, and the ‘Which is the Celebrity?’ task, respectively.

### 4.1. Same/Different Task Performance

**Figure 1:**
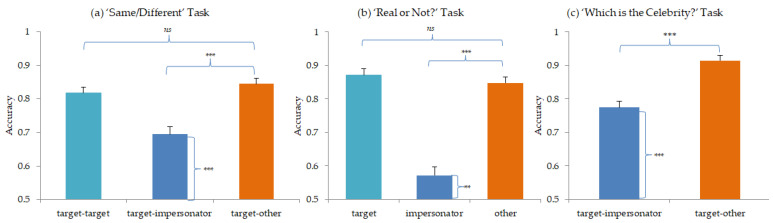
Mean accuracy (and standard error) in (**a**) the ‘Same/Different’ task, (**b**) the ‘Real or Not?’ task, and (**c**) the ‘Which is the Celebrity?’ task in Experiment 1 (*ns* indicates no significant difference; ** indicates a significant difference at *p* < 0.01; *** indicates a significant difference at *p* < 0.001).

Figure 1 summarises the data from all three tasks in Experiment 1. Within the ‘Same/Different’ task, the data suggested a better performance in the two easy conditions involving (i) the same speakers, and (ii) obviously different speakers than when discriminating between a target and their impersonator. Friedman’s ANOVA for repeated measures confirmed the main effect of the trial type (Friedman’s *Q*_(2)_ = 20.54, *p* < 0.001). Pairwise comparisons confirmed no significant difference in performance in the two easy conditions (target–target vs. target–other: *Q*_(1)_ = 1.09, *p* = 0.828). However there was a significant difference in ability when correctly saying ‘different’ in the easy condition and when correctly saying ‘different’ in the hard condition (target–other vs. target–impersonator: *Q*_(1)_ = 4.11, *p* < 0.001). This confirmed the intended difficulty when telling apart a target speaker from their impersonator.

Of most importance, a Wilcoxon one-sample comparison of performance in this hard condition relative to chance showed that the participants were able to tell apart the target from their impersonator at greater than chance levels (*W* = 1033.5, *p* < 0.001). This demonstrated that despite the naturally difficult task at hand, human listeners were nevertheless able to discriminate between two highly similar voices.

### 4.2. ‘Real or Not?’ Task Performance

As seen in Figure 1, the data within the ‘Real or Not?’ task suggested a better performance when judging the target to be ‘real’, and when judging the ‘matched other’ celebrity to be ‘not real’, than when reaching a decision for the impersonator voice. Friedman’s ANOVA for repeated measures confirmed this main effect of trial type (Friedman’s *Q*_(2)_ = 55.39, *p* < 0.001). Pairwise comparisons confirmed no significant difference in performance in the two easy conditions (target vs. other: *Q*_(1)_ = 0.343, *p* = 1.00). However, again, there was a large and significant difference in ability when saying ‘not real’ to the other celebrity than when saying ‘not real’ to the impersonator (*Q*_(1)_ = 5.93, *p* < 0.001).

As in the ‘Same/Different’ task, the question of greatest interest was whether the participants were still able to respond appropriately to the impersonator despite the difficulty of the task. A Wilcoxon one-sample comparison to chance demonstrated that whilst the task was hard, the participants were significantly better than guessing (*W* = 807.00, *p* = 0.009). Thus again, despite the naturally difficult task, human listeners were able to reject the impersonator as the named target.

### 4.3. ‘Which Is the Celebrity?’ Task Performance

The data summarised in Figure 1 suggest that the performance on the ‘Which is the Celebrity?’ task was far easier when the participants were asked to judge between the target and the ‘matched other’ voice than when asked to judge between the target and their impersonator. A Wilcoxon signed-rank test confirmed that the difference in the performance levels was significant (*W* = 808.50, *p* < 0.001).

In common with the previous two tasks, the question of importance was whether the participants were better than chance when presented with the target and their impersonator. A Wilcoxon one-sample comparison to the chance levels again demonstrated that whilst the task was difficult, the performance was nevertheless again significantly better than guessing (*W* = 1145.00, *p* < 0.001). Thus, the human listeners were able to be able to pick out the real target from their impersonator at better than chance levels of accuracy.

### 4.4. Comment on Prosodic and Acoustic Characteristics

The results above aligned with the predictions and highlighted the difficulty when telling apart vocal impersonators from their targets. In this regard, it was tempting to see whether the vocal impersonators were indeed a better likeness to the target than the ‘matched other’ voices.

A series of vocal characteristics was extracted using PRAAT 6.2.23 in order to describe each clip for each of the targets, impersonators, and ‘matched other’ speakers. The chosen characteristics were selected in order to reflect the influence of the vocal folds and vocal tract shape, both of which have been highlighted as influencing individual differences in voice production [19,31]. In addition, the selections were mindful of the vocal characteristics that the impersonators moderated in previous studies [26,27]. With these in mind, standard acoustic measures were utilised to reflect the vocal folds, namely, the fundamental frequency or ‘pitch’ (f0), variation in pitch or ‘intonation’ (f0 standard deviation), vocal hoarseness/noise (harmonics to noise ratio), and vocal turbulence reflected in the cycle-to-cycle pitch variation (jitter (local) and cycle-to-cycle amplitude variation (shimmer (local).

The measures usually used to capture the vocal tract shape commonly include the first four formants (F1–F4) from which the formant dispersion can be calculated [19]. However, given the different utterances in each speech clip, and the sensitivity of F1–F3 to these differences, we followed the recommendations highlighted by López et al. [30] and confined ourselves to the use of F4, which is more stable across utterances and thus more indicative of identity.

Once extracted, the values for each characteristic were then averaged across the clips for each identity in order to generate a single measure per characteristic and per identity for the purposes of comparison. Finally, standardised difference scores were generated for each characteristic, and were summed across all characteristics to provide an overall index of similarity (see Table 1).

Analysis of these vocal characteristics did not support the simple assumption that the impersonators would always be more similar to their celebrity targets compared to the ‘matched other’ voices. Taking these characteristics separately, a series of Wilcoxon signed-rank comparisons revealed no differences when comparing the target–impersonator similarity to the target–other similarity for all characteristics other than pitch (*W* > 29, *n* = 12, *p* > 0.433). The analysis of pitch did reveal a significant difference (*W* = 12.00, *n* = 12, *p* = 0.034). However, the data indicated that it was the ‘matched other’ speaker that was more similar to the target, and not the impersonator. Finally, when examining the overall index of similarity, the Wilcoxon signed-rank test again suggested no significant difference in target–impersonator similarity and target–other similarity (*W* = 26.00, *p* = 0.308).

Closer exploration of the overall similarity index in Table 1 revealed that the impersonator was more similar to the target than the ‘matched other’ voice for only five of the 12 targets (BJ, BC2, DA, GC, MF – indicated in bold in Table 1). This alone was perhaps notable given that the matched other voices were selected in order to provide a non-trivial point of comparison. Thus, they were all sex-matched speakers with similar pitch, accent, and age-range as judged by ear.

For the remaining impersonators, an examination of the measures highlighted several instances in which the impersonation reflected exaggeration rather than replication of the target on one or more of the measures, thus making the impersonations *less* similar to the target than the corresponding ‘matched other’ voice. This was particularly the case when examining pitch (i.e., EH) and melodic pitch contours, especially when the impersonator’s own voice could not physically approximate that of their intended target in other regards (i.e., PF and TM). The use of exaggeration or caricature by the impersonators in this study echoes that noted in previous analyses [26,27,28,29].

This aside, it is important to note that voices differ in different ways [1], and a single set of metrics is thus unlikely to capture all the cues that the impersonator may mimic or that the listener may perceive when processing identity. Additionally, it can be very difficult to find the acoustic correlates to describe the vocal similarities or differences that we hear as listeners [26]. Finally, we note that the present analysis did not capture the vocal elements associated with loudness, timing of speech (articulation rate) or of vowel sounds, and the paralinguistic or prosodic elements such as stutters, pauses, idiosyncratic speech rhythms, or patterns of emphasis. These characteristics have all been linked to simple judgements of speaker gender [32] as the well as more complex identification of a unique vocal identity. As such, the present acoustic analysis is offered to accompany the main analysis of listener performance, but it is noted that the acoustic analysis of voices will be limited by the characteristics selected for investigation, and that there is unlikely to be a single set of acoustic characteristics that can capture the idiosyncrasies of all voices.

## 5. Discussion—Experiment 1

The results of Experiment 1 suggest that despite the presentation of a very difficult natural listening challenge involving telling apart targets from their impersonators, the participants were better than chance across the three tasks. Specifically, they were above chance when concluding that the target and impersonator were ‘different’ (Task 1), when concluding that the impersonator was ‘not real’ (Task 2), and when picking out the target over the impersonator (Task 3).

This is not to say that the listeners were never fooled. Indeed, item analysis confirmed that more often than not, two of the twelve impersonators were mistakenly judged as the same person as their target (EH, RW) in Task 1; four impersonators were mistakenly selected as the ‘real’ named target (BC, BC2, CC, EH) in Task 2; and one impersonator was mistakenly identified rather than the target (EH) in Task 3. These results confirmed that the impersonations used in the current task were of high quality. Additionally, the fact that the impersonators chose semantic content that aligned with their target may have promoted a readiness [26] to accept the imitation and succumb to the dupe. This may have been particularly likely during the ‘Real or Not?’ task compared to the other two tasks because the ‘Real or Not?’ task depended on a comparison of a single clip to some stored mental representation whereas the ‘Same/Different’ task and the ‘Which is the Celebrity?’ task afforded some level of comparison across voice clips. The memory demand additionally rendered the ‘Real or Not?’ task vulnerable to any distortions of memory, and these are known to occur, particularly in terms of an exaggeration of vocal pitch [33]. Consequently, any exaggeration of pitch by the impersonator may align with an exaggeration of pitch in memory, thus leading to a false conclusion that the impersonator is ‘real’. It should not be a surprise then that the impersonator condition in the ‘Real or Not?’ task gave rise to the weakest level of performance overall.

This aside, the results here were effective in introducing highly challenging listening conditions, and yet were not effective in removing voice processing performance entirely. As such, in this difficult task, the limit of human listener capability was not reached, suggesting that voice processing is stronger than one might previously have considered. The present study, however, only examined half of the voice recognition task—the *telling apart* of two similar sounding speakers—commonly considered to be the easier of the two elements in voice recognition [23]. Accordingly, Experiment 2 examined the performance on a naturally difficult version of the other half of voice recognition—*telling together* two different sounding versions of the same speaker.

## 6. Experiment 2: Telling Together Voices When Singing and Speaking

The flexibility of the human voice is considerable [34]. For instance, we may unconsciously change our voice depending on who we are talking to, with over-articulation noted in elderly-directed speech [35] and a higher sing-song pitch noted in infant-directed speech [36]. Our voices also change depending on whether we are reading or conversing [37,38,39,40,41], and depending on our emotional state [42,43]. Slower changes may also occur in the voice across one’s lifespan, reflecting natural ageing [44], vocal strain [45], or health issues such as tobacco consumption [46,47]. Additionally, of course, we may purposefully change our voice to make ourselves sound younger or older, or to hide our identity (i.e., [42,48,49,50]). This variation in our vocal repertoire requires that we are able to spot the similarities between different instances of the same speaker if we are to successfully recognise them. Experiment 2 presents an extreme version of this *telling together* challenge by asking speakers to alter their voices considerably. We did this by asking them to sing.

The singing voice differs from the speaking voice in several perceptible ways that go beyond the norm. Changes include the introduction of vibrato [51], the alteration of pitch, pitch contour (melody), rhythm, and timing characteristics, and the alteration of characteristics associated with the vocal tract shape imposed by a lowering of the larynx or a change in the position of the tongue and lips [52]. As a result, notable examples exist including Susan Boyle and George Ezra, where the singing voice is remarkably different to the speaking voice. Consequently, one might question whether it is possible to match a singing clip with a speaking clip from the same person.

Two studies are pertinent to this question. First, Bartholomeus [53] used a 4-alternate-forced-choice task and demonstrated above-chance matching of two singing voices (63.7% accuracy), and above-chance matching of two speaking voices (79.4% accuracy). Notably, the performance was better in the latter case than in the former case, suggesting some difficulty when processing the identity of singers. However, Bartholomeus did not test the ability to match a singing voice with a speaking voice.

Peynircioğlu, Rabinovitz and Repice [54] did, however, address this question. Using 24 unfamiliar targets and a same/different matching task, they examined the ability to match two speaking voices, two singing voices, and a cross-modal speaking–singing pair of voices. Performance was reported as above chance in all conditions, but the task was more difficult in the cross-modal condition than in the two unimodal conditions. The performance also depended on the content of the clip, with cross-modal matching performance being better when the content involved words (61.5%) than when it involved mere vowel sounds (54.5%). The latter condition appeared to have been particularly challenging, perhaps due to the unfamiliarity of processing mere vowel sounds in a spoken clip. Nevertheless, performance in the former condition involving spoken and sung words did appear to indicate that matching was possible.

Experiment 2 seeks to replicate this result using a pairing task that is more akin to a sorting task than to a same/different matching task. Experiment 2 also probed the performance across familiar and unfamiliar targets in order to explore whether prior familiarity may offset the challenge imposed by the task. As noted by Johnson, McGettigan, and Lavan [55], the use of a pairs sorting task here rather than a more standard same/different matching task may be advantageous as it does not dictate the use of any specific strategies. Success on this task would signal that the listener can spot the similarities between different clips from the same speaker despite considerable vocal variation. In contrast, an inability to complete this task may signal that the limits of human vocal processing have finally been reached.

## 7. Materials and Methods—Experiment 2

### 7.1. Design

Participants took part in a voice pairing task in which the familiarity of stimuli (celebrity, unfamiliar) and difficulty of task (two speaking clips, singing and speaking clips) were varied. Note that the third possible condition involving matching of two singing clips was not included here. This decision reflected a desire to keep the task to a manageable length. Additionally, the task of pairing two speaking clips was considered a more commonplace task than that of pairing two singing clips, and this familiarity of task in the former case was considered important in establishing a meaningful baseline. As such, a 2 × 2 within-participants design was used. The more difficult (singing-speaking) pairing task always preceded the easier (speaking–speaking) task in order to reduce the possibility that performance in the easier task might facilitate performance in the harder task. Within each task, however, the order of celebrity and unfamiliar tasks was counterbalanced. The accuracy of performance on each pairing task represented the dependent variable.

### 7.2. Participants

A total of 40 participants (26 females, 14 males) took part on a volunteer basis or were recruited via a departmental participant scheme and took part in return for course credit. As in Experiment 1, the sample size was determined by availability, however, a post-hoc power calculation using G*Power 3.1.9.7 (with alpha set to 0.05) showed that our sample size was associated with a power of 0.989.

The participant ages ranged between 18 and 29 years (M = 21.03, SD = 2.13), reducing the risk of age-related hearing loss. All participants had normal or corrected-to-normal hearing as determined through self-report, and none had taken part in Experiment 1. They also reported a good knowledge of current popular singers in the UK and reported a lack of familiarity with the unfamiliar targets.

### 7.3. Materials

#### 7.3.1. Unfamiliar Voices

A total of 12 unfamiliar targets were used (six females, six males), with each providing three speaking clips and one singing clip. The targets were all British and were drawn either from the local University ‘Jazzmanix’ choir (*n* = 6) or from an available group of YouTube cover singers (*n* = 6) who had a small enough following to make it unlikely that their voices would be familiar. Singing clips were extracted from covers of popular songs that were either posted on YouTube or recorded for the purposes of this study using an Olympus VN-541PC digital recording with a low-cut filter for noise cancellation. Songs were all prominent within the UK Top 50 chart at the time of this study, and the singers were recorded against an instrumental backing track. Speaking clips were either extracted from YouTube conversation by the YouTube singers, or from non-sequential passages of conversations by the Jazzmanix singers.

In addition to these targets, a total of six unfamiliar English speakers (three females, three males) provided a single speaking clip. These acted as fillers in the pairing task. As with the Jazzmanix singers, the filler speaking clips were extracted from longer conversations and the recordings were gathered as described above.

#### 7.3.2. Celebrity Voices

A total of 12 celebrity singers were used (six females, six males), all of whom were popular British singers within the UK Charts at the time of testing. As above, each celebrity provided three speaking clips and one singing clip. Singing clips were extracted from purchased cover songs recorded against an instrumental backing track. The use of cover songs was considered important so that the song itself could not indicate the identity of the singer. Speaking clips were extracted from radio or chat-show interviews, with care taken to ensure that (i) the content of the speech did not reveal the identity of the speaker and (ii) the content of one speech clip did not follow on from the content of another.

Finally, as with the unfamiliar voice set above, a set of six British celebrity fillers (three females, three males) provided a single speaking clip from a radio or chat-show interview, with the content of speech not revealing identity as above.

#### 7.3.3. Editing of Clips

All speaking clips were edited using Audacity 3.1.0 to create audio clips of approximately eight seconds. All singing clips were edited to extract four bars of the singing voice, thus providing a sufficient sample to listen to regardless of the tempo of the song.

#### 7.3.4. Trial Construction

Four pair tasks were created consisting of (i) unfamiliar speaking–speaking pairs; (ii) celebrity speaking–speaking pairs; (iii) unfamiliar singing–speaking pairs; and (iv) celebrity singing–speaking pairs.

**Figure 2:**
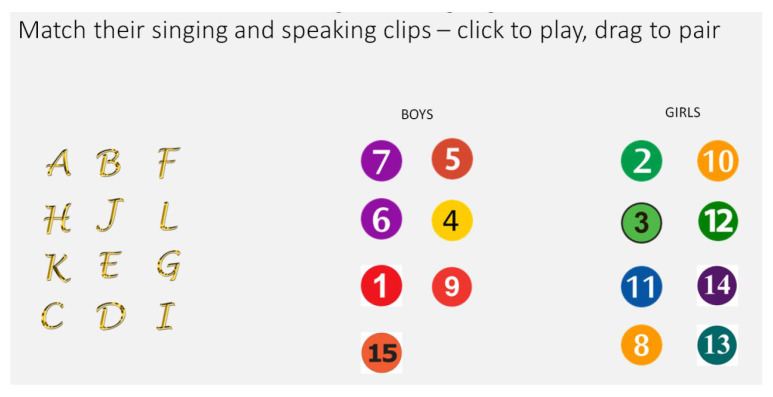
Screenshot showing the arrangement of icons at the start of a pairing trial in Experiment 2.

To create the speaking–speaking tasks, two speech clips for each of the 12 (celebrity or unfamiliar) targets were arranged on a PowerPoint slide. One set of speech clips was arranged on the left-hand side of the slide with each depicted by a letter (A to L). The second set of speech clips was arranged on the right-hand side of the slide with each depicted by a number (1 to 15 including three filler speech clips). Thus, the task was to drag the icons together to match a letter with a number in order to pair up the two speech clips from the same person. For ease, the numbered speech clips were separated into male and female groups (see Figure 2 for an illustration of the starting point of a trial).

To create the singing–speaking tasks, the set of singing clips for each of the 12 (celebrity or unfamiliar) targets was arranged on the left-hand side of the slide, with each depicted by a letter (A to L). As above, the speaking clips were arranged on the right-hand side of the slide with each depicted by a number (1 to 15 including three filler speech clips). Again, the numbered speaking clips were separated into male and female groups. Care was taken to ensure that the letters and numbers used for one identity on one slide did not correspond to those used for the same identity on the next slide. Thus, the participants could not facilitate their task by remembering previous letter–number combinations.

#### 7.3.5. Presentation of Stimuli

The task was presented to participants using PowerPoint running in edit mode so that the participants could interact with the icons. The slides were presented on a MacBook Pro with a 15” colour screen and a screen resolution of 1366 × 768 pixels. Sound was played via the computer speakers set to an audible but adjustable level.

### 7.4. Procedure

Following the provision of informed consent, the participants were tested individually and in-person. Participants were randomly allocated to one of two conditions that differed only in the order of presentation of unfamiliar and celebrity stimuli within the easy (speaking–speaking) tasks and the more difficult (singing–speaking) tasks. This aside, the procedure for each task was identical. On-screen instructions informed the participants that their task was to click on each icon in order to play the audio clip, and then drag the icons together on the PowerPoint slide in order to pair up the clips belonging to the same person. The experimenter demonstrated how to play and how to drag icons so that the participants were clear as to how to respond. Participants were aware of the unequal number of clips, and this ensured that the last few pairings could not be achieved by a mere process of the elimination of the remaining stimuli.

The participants were invited to take as much time as necessary to complete the task. The completion of all four trials took no more than 30–40 min, after which the participants were thanked and debriefed.

### 7.5. Data Management and Statistical Analyses

As in Experiment 1, the data were collected in accordance with ethical principles as laid out in the Declaration of Helsinki, and the anonymous summary datafiles can be downloaded from the Supplementary Materials.

Statistical analyses were conducted to test whether the challenge when pairing singing and speaking clips from the same person created an impossible task. Performance was evaluated for familiar and unfamiliar targets to further test whether familiarity mitigated the task difficulty. Preliminary analyses suggested that the accuracy scores did not deviate from a normal distribution (*D* > 0.127, *p* > 0.114). Thus, parametric analyses were used consisting of a repeated-measures Analysis of Variance (ANOVA), with *t*-tests for follow-up comparisons. As in Experiment 1, an alpha of 0.05 was assumed throughout, with Bonferroni-correction in the case of multiple comparisons.

## 8. Results—Experiment 2

The data from one participant were excluded from analysis due to a failure to complete all trials. This participant was removed and replaced. Analysis across participants also revealed very low performance for one participant in the celebrity speaking–speaking pairs task (accuracy = 17%), which identified this participant as an outlier (below 1^st^ quartile minus 1.5 × interquartile range). The data from this participant were removed from all remaining analyses, leaving the data for 39 participants.

**Figure 3:**
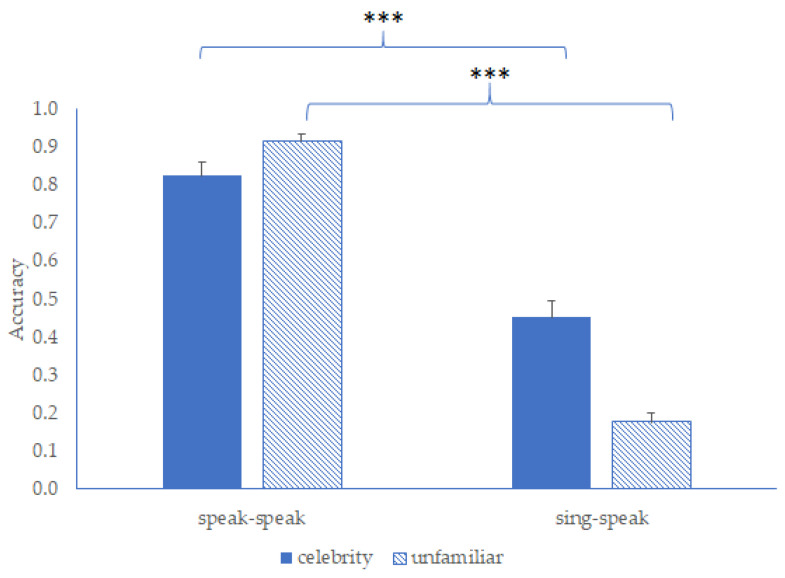
The mean accuracy of identity matching (and standard error) in the pairing task in Experiment 2 (*** indicates a significant difference at *p* < 0.001).

The accuracy of performance on the pairing task was calculated across the 12 celebrity targets and the 12 unfamiliar targets. This was used as a measure of performance in the easy condition (speaking–speaking) and the more difficult condition (singing–speaking). These data are summarised in Figure 3.

A 2 × 2 repeated-measures ANOVA was conducted in order to examine the effects of familiarity (celebrity, unfamiliar) and task difficulty (speaking–speaking, singing–speaking) on performance. This revealed a significant main effect of familiarity (*F*_(1, 38)_ = 7.98, *p* = 0.008, η^2^_p_ = 0.17) such that the performance was better with celebrity targets than with unfamiliar targets. In addition, it revealed a significant main effect of task difficulty (*F*_(1, 38)_ = 378.12, *p* < 0.001, η^2^_p_ = 0.91) with performance being better when pairing two speaking clips than when pairing a singing clip to a speaking clip. Finally, a significant interaction emerged (*F*_(1, 38)_ = 70.65, *p* < 0.001, η^2^_p_ = 0.65), which was examined by a series of Bonferroni-corrected repeated-measures *t*-tests.

The *t*-tests revealed that pairing performance was significantly affected by the task difficulty for both the celebrity voices (*t*_(38)_ = 8.88, *p* < 0.001, Cohen’s d = 1.42) and for unfamiliar voices (*t*_(38)_ = 25.55, *p* < 0.001, Cohen’s d = 4.09). However, the magnitude of the effect was far greater for unfamiliar voices than for celebrity voices (*t*_(38)_ = 8.41, *p* < 0.001, Cohen’s d = 1.35). As a result, these findings suggest that the pairing singing and speaking clips represented a difficult *telling together* task, as intended. However, echoing the results of Lavan, Burston et al. [25], familiarity with the speakers afforded the participants a greater tolerance of vocal change, making them more successful at this task.

Finally, in order to determine whether the participants had reached the limit of capability in this task, performance was examined in the difficult singing–speaking condition relative to zero and relative to chance. This belt-and-braces approach was taken given that the lack of specific strategies in the pairing task means that it does not lend itself to the concept of ‘chance level responding’ in the same way that a same/different discrimination task does. Indeed, the notion of chance level responding is complicated not least because a participant may choose to pair a voice with reference to some or all of the available candidates, but may also choose *not* to pair a voice at all. This said, a series of one-sample *t*-tests confirmed that when the voices were familiar, the matching of singing and speaking clips exceeded both zero level (*t*_(38_) = 10.05, *p* < 0.001, Cohen’s d = 1.61) and chance level performance *t*_(38)_ = 7.42, *p* < 0.001, Cohen’s d = 1.19). The same was true when the voices were unfamiliar: the matching of singing and speaking clips exceeded zero (*t*_(38)_ = 7.46, *p* < 0.001, Cohen’s d = 1.20) and exceeded chance level performance (*t*_(38)_ = 2.44, *p* = 0.020, Cohen’s d = 0.39). These results suggest that the current task of spotting similarities between different instances of the same voice approached but did not reach the limit of our capability.

### Confusability of Stimuli

**Figure 4:**
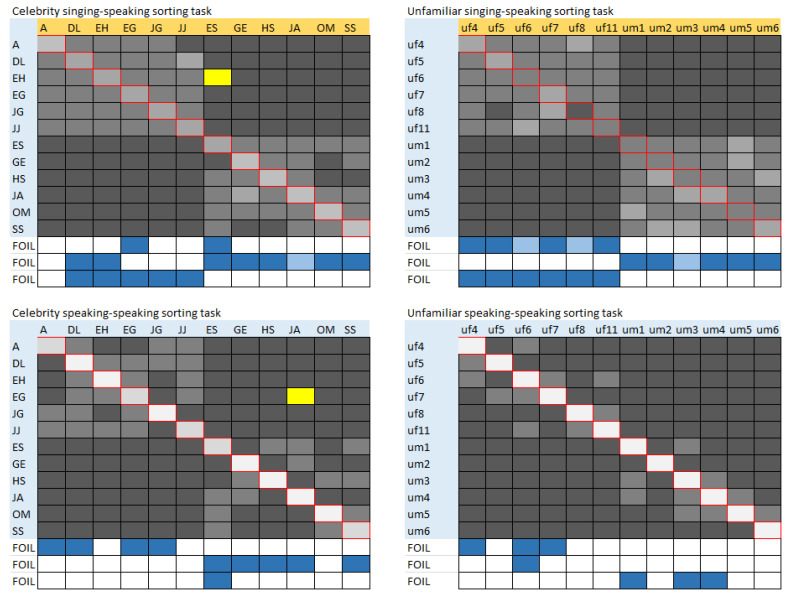
Confusability matrices when matching the singing and speaking clips (top two panels) and when matching the two speaking clips (bottom two panels). Celebrity matrices are shown on the left. Erroneous matches to the foil speaking clips are shown in blue in the bottom three rows of each matrix. Lighter cells indicate a higher incidence of pairing and thus are expected along the diagonal from top left to bottom right. Yellow cells indicate an erroneous cross-sex match.

The use of a pairing task enabled a more detailed look at the data by way of a confusability matrix (see Figure 4). Rather than a blunt and categorical determination of whether a pairing was correct or not, this confusability matrix allowed for visualisation of the voices that tended to be mistakenly paired with one another. A higher incidence of pairing was depicted by a lighter colour within the matrix and was thus expected along the diagonal from top left to bottom right, which represented the correct pairing of two clips from the same person. Echoing the results of the statistical tests, it was clear from these confusability matrices that the diagonal was lighter (accurate identity pairing was greater) when presented with two speaking clips (bottom row) than when presented with singing and speaking clips (top row). This reflected the difficulty of the singing manipulation. However, it was also clear that the diagonal when pairing celebrity singing and speaking clips (top left) was lighter than when pairing unfamiliar singing and speaking clips (top right) (in which the diagonal was barely discernible). This reflected the statistical demonstration of the fact that familiarity mitigated task difficulty.

The matrices also allowed visualisation of another interesting feature of performance. When considering the relatively easy task of pairing of two speaking clips in Figure 3, the overall performance data indicated surprisingly better performance for the unfamiliar voices than for the celebrity voices (*t*_(38)_ = 2.89, *p* = 0.006, Cohen’s d = 0.463). The confusability matrices echoed this, showing less confusion with other voices in the unfamiliar case (bottom right—26 identities confused) than in the celebrity case (bottom left—44 identities confused). Moreover, the yellow-coloured cells indicated a male/female confusion, and this only occurred with celebrity voices.

A cautious interpretation of this unanticipated aspect of performance was that the increased familiarity with a voice enabled the development of a stored mental representation that captured the variety of instances experienced. This may have enabled the listener to better appreciate variation in the familiar voice, thus enabling better pairing of the singing and speaking clips. However, the tolerance to variability in the familiar speaker may have also resulted in the misinterpretation of a modest level of between-person variability as within-person variability, hence the errors made. The same was not true for unfamiliar voices where the rapidly formed mental representation was based on a single voice clip and thus did not capture the vocal variability at all. Pairing singing and speaking clips was thus very difficult, and yet pairing the two speaking clips was better than anticipated, noting that the two speaking clips used in this experiment were likely to have varied only a little.

## 9. Discussion—Experiment 2

Experiment 2 provided listeners with a naturally difficult sorting task by asking them to pair a singing clip with a speaking clip from the same person. Performance on this task was compared to the more standard task when pairing two speaking clips from the same person. Moreover, performance on this task was evaluated when the speakers were unfamiliar to the listener and when well-known.

The results suggest that performance was better when pairing two speaking clips than when pairing a singing clip with a speaking clip. This supported our expectations given that the singing voice represented a substantial variation from the normal speaking voice. Moreover, and as expected, performance was better when the voices were known to the listener than when unknown. Of more interest, the familiarity of the voice enabled the listener to cope better with the variation caused by singing. These results suggest a benefit from having an existing stored mental representation that likely reflected the natural variation that listeners had already experienced in each known voice. This particularly helped the listener when making sense of extreme albeit natural vocal change brought about by singing. Interestingly, however, this tolerance of vocal change in the familiar voice may have contributed to a misinterpretation of the between-person variability as within-person variability, thus causing a greater instance of pairing errors when listening to different celebrity speakers. This was an unanticipated finding, which remains an interesting element for future work to explore.

This aside, the results of Experiment 2 revealed that despite the difficulty associated with spotting similarities across singing and speaking clips from the same person, the performance remained significantly above zero and significantly above chance. As such, we again did not reach the limit of our capability.

## 10. General Discussion

The intention behind the current paper was to answer two questions. First, what is the point at which we fail to tell apart two similar sounding speakers; and second, what is the point at which we fail to tell together two different sounding instances of the same speaker? The two experiments reported here addressed these questions by providing extremely difficult yet natural challenges for the listener in the form of spotting differences between two speakers who sound very similar (Experiment 1) and spotting similarities within a single speaker who sounds very different (Experiment 2). The results followed all predictions, whilst also revealing some unanticipated effects. Specifically, whilst our natural challenges were successful in introducing difficulty to each task, performance remained above the level of mere guessing in every task and thus our listeners did not fail either to tell apart, or to tell together the voices we presented. This is not to say that their performance was free from mistakes. Certainly, some impersonators fooled some listeners, and one unfamiliar singing and speaking pair could not be paired at all. However, the overall performance remained above chance, implying that the current manipulations had not taken our listeners to the limits of their capability. Thus, the two questions that inspired this work cannot be answered yet.

Taking the results as a whole, these studies have provided an interesting and effective set of natural challenges for the human listener. The results lead to the conclusion that when pressed to respond to significant natural listening challenges, we are capable of resolving very high levels of within-speaker variability and very low levels of between-speaker variability. The processing of vocal identity thus appears to be far more sophisticated than we might previously have considered. The current conclusions relate specifically to the listening skills of those drawn from the normal population, and separate studies of participants who occupy the extremes of phonagnosia or super-recogniser status may come next. However, for now, it has to be concluded that we have simultaneously failed to define our voice processing limits, and yet we have revealed our considerable voice processing strengths.

Two aspects of the present results are, however, of more general interest and warrant further consideration.

### 10.1. Regions in Voice Space Offer a Helpful Metaphor

The first observation that we draw from the current results is an appreciation of the value of considering identity representations as regions rather that points in identity space. Several authors have discussed this concept (see [21,56,57]), and here we note the twin benefits that follow from this perspective. First, the recognition task can be understood as the successful mapping of an exemplar into the identity region of a known person, and this overcomes the awkward alternative that involves mapping onto a point in space with which there might be an imperfect correspondence. Second, and somewhat related, conceiving of an identity representation as a region rather than a point in space allows for explicit acknowledgement of the fact that exemplars within an identity will naturally vary, and this facilitates the theoretical discussions around spotting similarities within a person as well as spotting differences between people.

### 10.2. Familiarity Informs an Appreciation of Individual Vocal Variability

The second observation follows from the previous one and reflects on the influence of familiarity in reducing the impact of task difficulty when telling voices together. Viewing mental representations as regions within an identity space provides a natural and ready explanation for this familiarity effect. Indeed, one can consider that as a voice becomes more and more familiar through repeated exposure, the range of instances experienced will inform an appreciation of how, and by how much, that voice can vary. This links with what Vernon (cited in [58] (p. 8)) described as the ‘possible and permissible permutations’ within an identity, allowing reference to the meaningful variability that an individual may display across different moments in time (see [24]). It should not be surprising, therefore, that we are better able to resolve intra-speaker variability when we have already been exposed to intra-speaker variability. The fact that we can resolve intra-speaker variability at all for unfamiliar or once-heard voices may be explained by the extraction of general rules regarding how and by how much a standard voice may vary. However, knowing how a *particular* voice varies will always provide a greater advantage than knowing how a general voice varies, hence the familiarity effects observed.

This said, it was through consideration of the familiarity effects in Experiment 2 that a surprising result emerged. This related to the greater incidence of mistaken pairings when matching two celebrity speaking clips than when matching two unfamiliar speaking clips. This finding emerged when looking at both the overall accuracy of pairing performance and when considering the sheer number of celebrity identities that were confused. Our interpretation of this unexpected finding suggests that the stored mental representations for familiar voices will, by their nature, capture the variability and enable a tolerance to vocal change. Whilst this can help when resolving within-person variability, it can also result in the misinterpretation of between-person variability as within-person variability, resulting in the confusion errors seen. In contrast, tolerance to variability in the unfamiliar voice will be reduced, given that variability in that voice has not been experienced, and thus the listener may demand that pairings are more exact, with fewer confusions as a result. This aspect of the current findings is intriguing, given that it was so unanticipated. Certainly, future work is encouraged to see whether the effect is replicated.

## 11. Conclusions and Final Thoughts

The present paper set about defining the limits of our ability to spot similarities in voices from the same person and to spot differences in voices from different people. Through two experiments that introduced considerable natural listening challenges, we failed to show a breakdown in task performance, and instead showed that the voice processing capability of the normal listener is rather exceptional and is likely far better than previously anticipated. Importantly, the ability to resolve within-speaker variability was improved by the familiarity of the stimuli. This can be explained when stored mental representations are viewed as regions that capture and reflect the vocal variability within a speaker. From this basis, two final interesting questions become apparent.

The first is a natural extension to the above discussion of familiarity, and it concerns the extent of familiarity required to elicit the familiarity benefits observed in Experiment 2. This may now perhaps be restated as the extent of familiarity required to form a robust representation capable of enabling the interpretation of within- and between-speaker variability. The answer to this question may be trivial, in that there may exist a continuum through from unfamiliar voices, once heard voices, lab-learned voices, celebrity voices, and personally familiar voices, through to family, friends, and one’s own voice. Indeed, a growing exposure to vocal variety may be associated with a growing robustness of representation [59] and a growing benefit when both *telling together* and *telling apart* instances of familiar speakers. Nevertheless, empirical work has not yet been conducted to determine whether increasing levels of familiarity with a speakers’ voice can be revealed in tasks such as the sorting task. Given the sensitivity of the sorting task to subtle aspects of voice identity performance, this seems like a valuable question to pursue, and the sorting task seems to be a valuable methodology to use. Future work is eagerly awaited in this vein.

Finally, we reflect on the question of individual differences between speakers. Already noted are instances within the current work where the impersonator successfully fooled our listeners, and where a particular singer could not be matched with their speaking voice. These highlight the fact that voices differ in how they differ. Indeed, Burton [60]) suggested that the type and extent of variation in a (face or) voice may itself be a marker of identity. Existing work is still perhaps stuck on considering the item effects as something to statistically control for or design out through the use of larger stimulus sets. However, in the same way that the field has now come to view intra-individual variability as something that is interesting in and of itself, we have come to view item effects as being equally worthy of our attention. Exciting next steps could begin to explore the extent to which the characteristics of a voice dictate and determine its variability, and thus its recognisability. Such explorations would augment the existing view of mental representations of identity, contributing meaningfully towards a more complete description of our unique sonic signature.

## Figures and Tables

**Table 1:** Summed standardised difference scores when comparing the acoustic voice measures of the targets with those of the impersonators and ‘matched other’ celebrities. Note that the summed standardised scores represent the combined differences in fundamental frequency (F0), pitch variability (F0 standard deviation), vocal tract shape (F4), vocal turbulence (jitter (local), shimmer (local)), and hoarseness (harmonics to noise ratio). Bold text indicates the celebrity targets for whom the impersonator is more similar to the target than is the ‘matched other’.

Celebrity Target	Target vs. Impersonator	Target vs. Other
BC	13.29	8.41
**BJ**	**6.24**	9.21
**BC2**	**6.19**	6.44
CC	11.64	6.24
**DA**	**5.78**	8.71
EH	4.03	2.77
**GC**	**5.22**	12.14
**MF**	**6.71**	7.51
PF	8.79	5.42
RG	10.38	2.75
RW	11.66	5.94
TM	8.9	8.63

## Data Availability

The data presented in this study are available in the Supplementary Materials.

## Supplementary Materials

The following supporting information can be downloaded as excel files at: https://www.mdpi.com/article/10.3390/brainsci13020358/s1. Experiment 1_impersonator performance; Experiment 2_singer performance.

## References

[1] Kreiman J., Sidtis D. (2011). Foundations of Voice Studies: An Interdisciplinary Approach to Voice Production and Perception.

[2] Frühholz S., Belin P. (2019). The Oxford Handbook of Voice Perception.

[3] Stevenage S.V., Neil G.J. (2014). Hearing Faces and Seeing Voices: The Integration and Interaction of Face and Voice Processing. Psychol. Belg..

[4] Belin P., Fecteau S., Bédard C. (2004). Thinking the voice: Neural correlates of voice perception. Trends Cogn. Sci..

[5] Belin P., Bestelmeyer P.E.G., Latinus M., Watson R. (2011). Understanding Voice perception. Br. J. Psychol..

[6] Young A.W., Frühholz S., Schweinberger S.R. (2020). Face and voice perception: Understanding commonalities and differences. Trends Cogn. Sci..

[7] Ellis H.D., Jones D.M., Mosdell N. (1997). Intra- and Inter-Modal Repetition Priming of Familiar Faces and Voices. Br. J. Psychol..

[8] Hanley J.R., Smith S.T., Hadfield J. (1998). I recognize you but can’t place you. An Investigation of Familiar-Only Experiences during Tests of Voice and Face Recognition. Q. J. Exp. Psychol. Sect. A.

[9] Barsics C., Brédart S. (2011). Recalling episodic information about personally known faces and voices. Conscious. Cogn..

[10] Brédart S., Barsics C. (2012). Recalling semantic and episodic information from faces and voices: A face advantage. Curr. Dir. Psychol. Sci..

[11] Damjanovic L., Hanley J.R. (2007). Recalling episodic and semantic information about famous faces and voices. Mem. Cogn..

[12] Barsics C., Brédart S. (2012). Recalling semantic information about newly learned faces and voices. Memory.

[13] Brédart S., Barsics C., Hanley R. (2009). Recalling semantic information about personally known faces and voices. Eur. J. Cogn. Psychol..

[14] Hanley J.R., Damjanovic L. (2009). It is more difficult to retrieve a familiar person’s name and occupation from their voice than from their blurred face. Memory.

[15] Cook S., Wilding J. (1997). Earwitness Testimony 1: Voices, Faces and Context. Appl. Cogn. Psychol..

[16] Stevenage S.V., Howland A., Tippelt A. (2011). Interference in Eyewitness and Earwitness Recognition. Appl. Cogn. Psychol..

[17] Stevenage S.V., Neil G.J., Hamlin I. (2014). When the face fits: Recognition of celebrities from matching and mismatching faces and voices. Memory.

[18] Tomlin R.J., Stevenage S.V., Hammond S. (2016). Putting the pieces together: Revealing face-voice integration through the facial overshadowing effect. Vis. Cogn..

[19] Baumann O., Belin P. (2010). Perceptual Scaling of Voice Identity: Common Dimensions for Different Vowels and Speakers. Psychol. Res..

[20] Valentine T. (1991). A unified account of the effects of distinctiveness, inversion and race in face recognition. Q. J. Exp. Psychol. Sect. A.

[21] Lavan N., Burton A.M., Scott S.K., McGettigan C. (2019). Flexible voices: Identity perception from variable vocal signals. Psychon. Bull. Rev..

[22] Jenkins R., White D., Van Montfort X., Burton A.M. (2011). Variability in photos of the same face. Cognition.

[23] Lavan N., Burston L.F.K., Garrido L. (2018). How many voices did you hear? Natural variability disrupts identity perception from unfamiliar voices. Br. J. Psychol..

[24] Stevenage S., Symons A., Fletcher A., Coen C. (2019). Sorting through the impact of familiarity when processing vocal identity: Results from a voice sorting task. Q. J. Exp. Psychol..

[25] Lavan N., Burston L.F.K., Ladwa P., Merriman S.E., Knight S., McGettigan C. (2019). Breaking voice identity perception: Expressive voices are more confusable for listeners. Q. J. Exp. Psychol..

[26] Zetterholm E. Same speaker–different voices. A study of one impersonator and some of his different imitations. Proceedings of the 11st Australian International Conference of Speech Science and Technology.

[27] Kitamura T. Acoustic analysis of imitated voice produced by a professional impersonator. Proceedings of the INTERSPEECH.

[28] Zetterholm E. The same but different—Three impersonators imitate the same target voices. Proceedings of the 15th International Congress of Phonetic Science.

[29] Laver J. (1994). Principles of Phonetics.

[30] López S., Riera P., Assaneo M.F., Eguía M., Sigman M., Trevisan M.A. (2013). Vocal caricatures reveal signatures of speaker identity. Sci. Rep..

[31] Roswandowitz C. Do humans distinguish deepfake from real vocal identity? Insights from the perceptual and neurocognitive system. Proceedings of the Presentation at the FaceID Conference.

[32] Leung Y., Oates J., Chan S.P. (2018). Voice articulation and prosody contribute to listener perceptions of speaker gender: A systematic review and meta-analysis. J. Speech Lang. Hear. Res..

[33] Mullennix J.W., Stern S.E., Grounds B., Kalas R., Flaherty M., Kowalok S., May E., Tessmer B. (2010). Earwitness memory: Distortions for voice pitch and speaking rate. Appl. Cogn. Psychol..

[34] Bin Amin T., Marziliano P., German J.S. Nine voices, one artist: Linguistic and acoustic analysis. Proceedings of the Conference Paper at IEEE International Conference on Multimedia and Expo (ICME).

[35] Kemper S., Finter-Urczyk A., Ferrell P., Harden T., Billington C. (1998). Using elderspeak with older adults. Discourse Process..

[36] Panneton Cooper R., Abraham J., Berman S., Staska M. (1997). The development of infants’ preference for motherese. Infant Behav. Dev..

[37] Hazan V., Baker R. Does reading clearly produce the same acoustic-phonetic modifications as spontaneous speech in a clear speaking style?. Proceedings of the DiSS-LPSS Joint Workshop 2010.

[38] Lavan N., Scott S.K., McGettigan C. (2016). Impaired generalisation of speaker identity in the perception of familiar and unfamiliar voices. J. Exp. Psychol. Gen..

[39] Guldner S., Lally C., Lavan N., Wittmann L., Nees F., Flor H., McGettigan C. Human Talkers Change Their Voices to Elicit Specific Trait Percepts, 28 November 2022. https://scholar.google.co.uk/scholar_url?url=https://psyarxiv.com/afky7/download&hl=en&sa=X&d=11251864003234394539&ei=b1KVY4SbC4r5yATy25i4Aw&scisig=AAGBfm0CjD80IiqyQJbqAFl8Jo1ubXtrsw&oi=scholaralrt&hist=g5lhrbQAAAAJ:13405656015283593831:AAGBfm3RxXSeZX2j-8amJkHzlKgFK1tNFw&html=&pos=0&folt=art.

[40] Scott S., McGettigan C. (2016). The voice: From identity to interactions. APA Handbook of Nonverbal Communication.

[41] Smith H.M.J., Baguley T.S., Robson J., Dunn A.K., Stacey P.C. (2018). Forensic voice discrimination by lay listeners: The effect of speech type and background noise on performance. Appl. Cogn. Psychol..

[42] Clifford B.R. (1980). Voice identification by human listeners: On earwitness reliability. Law Hum. Behav..

[43] Salsove H., Yarmey A.D. (1980). Long-term auditory memory: Speaker identification. J. Appl. Psychol..

[44] Linville S.E. (1996). The sound of senescence. J. Voice.

[45] Williams N.R. (2003). Occupational groups at risk of voice disorders: A review of the literature. Occup. Med..

[46] Damborenea T.J., Fernández L.R., Llorente A.E., Naya G.M., Marín G.C., Rueda G.P., Ortiz G.A. (1999). The effect of tobacco consumption on acoustic voice analysis. Acta Otorrinolaringol. Esp..

[47] Sorensen D., Horii Y. (1982). Cigarette smoking and voice fundamental frequency. J. Commun. Disord..

[48] Orchard T.L., Yarmey A.D. (1995). The effects of whispers, voice sample duration and voice distinctiveness on criminal speaker identification. Appl. Cogn. Psychol..

[49] Pollack I., Pickett J.M., Sumby W.H. (1954). On the identification of speakers by voice. J. Acoust. Soc. Am..

[50] Reich A.R., Moll K.L., Curtis J.F. (1976). Effects of selected vocal disguises upon spectrographic speaker identification. J. Acoust. Soc. Am..

[51] Sundberg J. (1977). The acoustics of the singing voice. Sci. Am..

[52] Künzel H.J. (2000). Effects of voice disguise on speaking fundamental frequency. Int. J. Speech Lang. Law.

[53] Bartholomeus B. (1974). Dichotic singer and speaker recognition. Bull. Psychon. Soc..

[54] Peynircioğlu Z., Rabinovitz B., Repice J. (2017). Matching Speaking to Singing Voices and the Influence of Content. J. Voice.

[55] Johnson J., McGettigan C., Lavan N. (2020). Comparing unfamiliar voice and face identity perception using identity sorting tasks. Q. J. Exp. Psychol..

[56] Lewis M.B., Johnston R.A. (1999). A unified account of the effects of caricaturing faces. Vis. Cogn..

[57] Stevenage S.V. (2018). Drawing a distinction between familiar and unfamiliar voice processing: A review of neuropsychological, clinical and empirical findings. Neuropsychologia.

[58] Bruce V. (1994). Stability from variation: The case of face recognition. MD Vernon memorial lecture. Q. J. Exp. Psychol. Sect. A.

[59] Tong F., Nakayama K. (1999). Robust representations for faces: Evidence from visual search. J. Exp. Psychol. Hum. Percept. Perform..

[60] Burton A.M. (2013). Why has research in face recognition progressed so slowly? The importance of variability. Q. J. Exp. Psychol..

