# Supplementary material for: The Curious Case of Impersonators and Singers: Telling Voices Apart and Telling Voices Together under Naturally Challenging Listening Conditions
Source: Brain Sci. 2023 Feb 19;13(2):358. doi: 10.3390/brainsci13020358 (PMC9954053; doi:10.3390/brainsci13020358)
Supplement: Supplementary file 1 [file brainsci-13-00358-s001.zip › brainsci-2139600-supplementary.pdf]

Experiment 1_impersonator performance

Participant	Same/Different Task - accuracy			Real or Not Task - accuracy		
	**target-target**	**target-impersonator**	**target-other**	**Target**	**Impersonator**	**Other**
p1	0.75	0.83	0.75	1	0.33	0.67
p2	0.67	0.75	0.92	0.83	0.67	0.92
p3	0.83	0.75	0.83	1	0.5	0.83
p4	1	0.75	0.92	1	0.42	1
p5	0.83	0.75	0.92	0.83	0.67	1
p6	0.67	0.67	0.83	0.83	0.67	0.75
p7	0.92	0.83	1	0.92	0.75	1
p8	0.92	0.67	0.75	0.67	0.67	0.92
p9	0.92	0.75	0.92	0.92	0.67	1
p10	0.75	0.5	0.92	1	0.42	0.83
p11	0.67	0.58	0.75	0.83	0.83	0.83
p12	1	0.5	0.83	0.92	0.33	1
p13	0.75	0.92	0.92	0.92	0.75	1
p14	0.58	0.92	0.92	1	0.58	0.75
p15	1	0.67	0.67	1	0.5	0.75
p16	0.75	0.58	0.75	0.92	0.33	0.75
p17	0.58	0.42	0.83	0.75	0.33	0.67
p18	0.58	1	0.92	0.67	0.92	1
p19	0.75	0.75	1	1	0.25	0.75
p20	0.92	0.67	0.67			
p21	0.58	0.58	0.83	0.92	0.42	0.83
p22	0.92	0.58	1	0.83	1	1
p23	0.75	0.5	1	0.92	0.42	0.83

p24	0.92	0.33	0.83	0.83	0.33	1
p25	0.92	0.42	0.83	0.92	0.75	0.83
p26	0.92	0.83	0.83	0.58	0.58	0.5
p27	0.75	0.92	0.83	1	0.58	0.83
p28	0.83	0.83	1	1	0.17	0.92
p29	0.5	0.75	0.67	0.67	0.58	0.67
p30	0.75	0.5	0.5	0.67	0.42	0.75
p31	0.92	0.67	0.83	1	0.58	0.92
p32				0.92	0.67	0.67
p33	0.92	0.42	0.83	0.92	0.42	0.58
p34	0.83	0.67	0.58	0.58	0.58	0.83
p35	0.92	0.5	0.92	0.92	0.5	0.92
p36	0.92	0.92	1	1	0.83	1
p37	0.92	0.83	0.92	1	0.42	1
p38	0.75	0.92	0.92	0.83	0.75	1
p39	0.75	0.92	0.75	0.58	0.58	0.75
p40	outlier on this task			0.75	0.33	0.5
p41	0.83	0.67	0.92	0.75	0.75	0.83
p42	0.92	0.33	0.75	outlier on this task		
p43	0.92	0.75	0.92	0.92	0.42	0.83
p44	0.92	0.58	0.92	0.83	0.67	0.75
p45	0.92	0.83	0.92	1	0.5	0.92
p46	0.67	0.75	0.83	1	0.83	0.83
p47	0.67	0.92	0.67	0.83	0.92	0.92
p48	0.75	0.83	1	1	0.67	1
p49	0.92	0.58	0.67	0.67	0.75	0.92
p50	1	0.67	0.83	1	0.67	0.83
p51	0.75	0.83	1	0.83	0.58	0.92

p52	0.92	0.75	0.67	0.92	0.58	0.83
p53	0.92	0.58	0.92	1	0.5	1
p54	outlier on this task			0.75	0.42	0.75

Experiment 2_singer performance.

Participant	Celebrity stimuli - accuracy of pairing			Unfamiliar Stimuli - accuracy of pairing	
	speaking-speaking	singing-speaking		speaking-speaking	singing-speaking
p1	1	0.42		1	0
p2	0.75	0.17		0.92	0
p3	1	1		1	0.5
p4	1	0.5		1	0.17
p5	1	0.42		0.92	0
p6			outlier		
p7	1	0.5		1	0.33
p8	0.58	0.08		0.92	0.17
p9	0.42	0		0.58	0.25
p10	1	0.17		1	0.08
p11	0.92	0.08		0.83	0.17
p12	0.92	0.67		1	0.17
p13	1	0.25		1	0.17
p14	0.67	0.17		1	0.25
p15	0.67	0.42		0.92	0.08
p16	1	0.75		1	0.25
p17	1	1		1	0.08

p18	0.83	0.42	1	0
p19	1	0.5	0.92	0.17
p20	1	0.58	1	0.58
p21	1	0.5	1	0.25
p22	0.5	0.75	0.92	0.25
p23	1	1	0.83	0.42
p24	0.67	0.17	0.83	0.42
p25	0.5	0.33	1	0.08
p26	1	1	0.83	0.08
p27	1	0.5	1	0.42
p28	0.83	0.5	1	0.08
p29	1	0.33	1	0
p30	did not complete all trials			
p31	0.58	0.42	0.67	0
p32	1	0.17	0.83	0.08
p33	0.25	0.17	0.83	0.33
p34	1	0.58	0.83	0.17
p35	0.58	0.58	0.67	0.08
p36	1	0.83	1	0.17
p37	0.83	0.33	0.75	0.17
p38	0.92	0.75	0.83	0.17
p39	0.67	0.17	1	0.08
p40	0.67	0.25	1	0
p41	0.33	0.17	0.83	0.17

Familiar singing-speaking sorting task

	A	DL	EH	EG	JG	JJ	ES	GE	HS	JA	OM	SS

A	0.59	0.18	0.05	0.03	0.13							
DL	0.05	0.31	0.08	0.08	0.13	0.23						
EH	0.05	0.08	0.38	0.18	0.05	0.13	0.03					
EG	0.08	0.13	0.18	0.33	0.10	0.08						
JG	0.15	0.18	0.08	0.08	0.28	0.08						
JJ	0.08	0.03	0.15	0.18	0.18	0.33						
ES							0.38	0.03	0.18	0.08	0.18	0.13
GE								0.56	0.03	0.05		0.15
HS							0.13	0.13	0.59	0.10		
JA							0.03	0.23	0.03	0.49	0.03	0.05
OM							0.10	0.03	0.05	0.05	0.54	0.05
SS							0.15			0.03	0.10	0.59
FOIL				0.05			0.03					
FOIL		0.03	0.03				0.13	0.03	0.10	0.21	0.15	0.03
FOIL		0.08	0.05	0.08	0.10	0.13						

Familiar speaking speaking sorting task

	A	DL	EH	EG	JG	JJ	ES	GE	HS	JA	OM	SS
A	0.77	0.05			0.08	0.08						
DL		0.82	0.03	0.08	0.03	0.05						
EH		0.03	0.90	0.03		0.05						
EG		0.03	0.03	0.72		0.13				0.03		
JG	0.05	0.03		0.03	0.85							
JJ	0.15	0.03	0.05	0.08		0.69						
ES							0.74		0.03	0.05		0.08
GE								0.92		0.03		
HS								0.03	0.92		0.03	0.03
JA							0.03	0.03		0.87		

	uf4	uf5	uf6	uf7	uf8	uf11	um1	um2	um3	um4	um5	um6
OM							0.05				0.92	0.03
SS							0.05				0.05	0.74
FOIL	0.03	0.03		0.05	0.05							
FOIL							0.05	0.03	0.05	0.03		0.10
FOIL							0.03					

Unfamiliar singing speaking sorting task

	uf4	uf5	uf6	uf7	uf8	uf11	um1	um2	um3	um4	um5	um6
uf4	0.23	0.03	0.05	0.13	0.26	0.13						
uf5	0.03	0.38	0.08	0.05	0.05	0.08						
uf6	0.13	0.15	0.15	0.13	0.08	0.15						
uf7	0.15	0.03	0.05	0.21	0.08	0.08						
uf8	0.08		0.03	0.23	0.00	0.08						
uf11	0.13	0.08	0.26	0.13	0.15	0.18						
um1							0.15	0.03	0.10	0.13	0.21	0.03
um2							0.08	0.10	0.10	0.10	0.28	0.08
um3							0.10	0.26	0.10	0.18	0.08	0.23
um4							0.13	0.10	0.23	0.26	0.08	0.15
um5							0.28	0.10	0.03	0.10	0.10	0.13
um6							0.10	0.23	0.21	0.08	0.10	0.23
FOIL	0.08	0.13	0.26	0.08	0.23	0.18						
FOIL							0.15	0.18	0.23	0.15	0.13	0.13
FOIL	0.15	0.18	0.13	0.05	0.15	0.10						

Unfamiliar speaking speaking sorting task

	uf4	uf5	uf6	uf7	uf8	uf11	um1	um2	um3	um4	um5	um6
uf4	0.90		0.05									
uf5	0.03	0.97										

uf6	0.03		0.82	0.05		0.03						
uf7		0.03	0.03	0.92								
uf8					0.97	0.03						
uf11			0.03		0.03	0.95						
um1							0.90		0.03			
um2								1.00				
um3							0.05		0.82	0.10		
um4							0.03		0.03	0.82	0.03	
um5									0.03	0.03	0.97	0.03
um6												0.97
FOIL	0.05		0.03	0.03								
FOIL			0.05									
FOIL							0.03		0.10	0.03		
